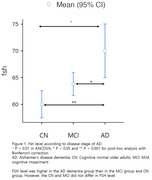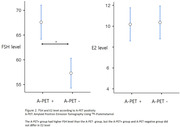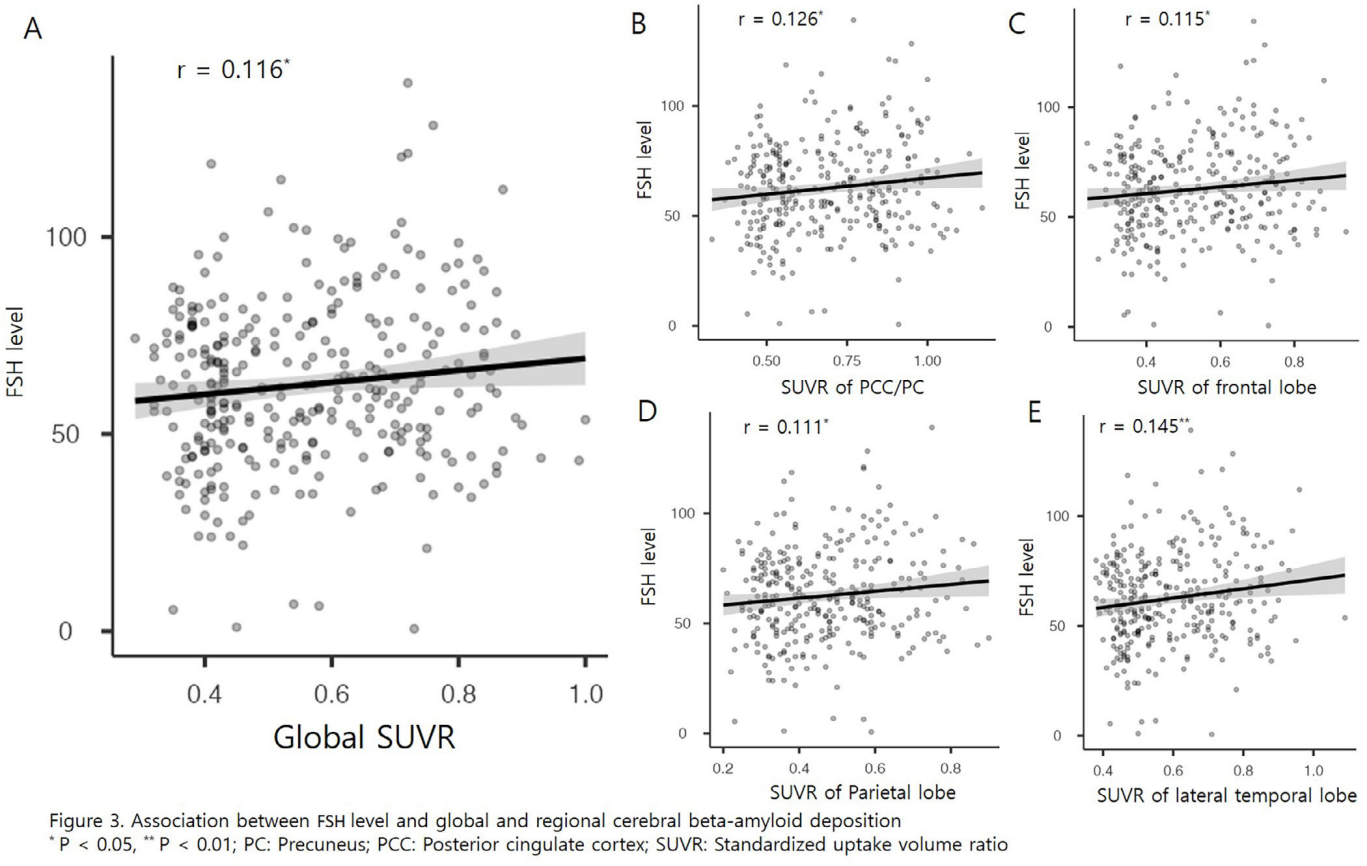# Differential Association of Follicle‐Stimulating Hormone and Estrogen with Disease Severity and Cerebral Amyloid Deposition in Alzheimer’s Disease Spectrum

**DOI:** 10.1002/alz.088504

**Published:** 2025-01-03

**Authors:** Sheng‐Min Wang, Chaiho Jeong, Yoo Hyun Um, Dong Woo Kang, Sunghwan Kim, Chang Uk Lee, Ki‐Hyun Baek, Hyun Kook Lim

**Affiliations:** ^1^ College of Medicine, Yeouido St. Mary’s Hospital, The Catholic University of Korea, seoul, seoul Korea, Republic of (South); ^2^ Medical College, The Catholic University of Korea, Seoul, Korea Korea, Republic of (South); ^3^ St. Vincent' Hospital, College of Medicine, The Catholic University of Korea, Suwon, Gyeonggi‐do Korea, Republic of (South); ^4^ College of Medicine, Seoul St. Mary’s Hospital, The Catholic University of Korea, Seoul, Seoul Korea, Republic of (South); ^5^ Yeouido St. Mary’s Hospital, College of Medicine, The Catholic University of Korea, Seoul, Korea Korea, Republic of (South); ^6^ Seoul St. Mary’s hospital, Catholic Medical College, the Catholic University of Korea, Seoul Korea, Republic of (South); ^7^ The Catholic University of Korea, Seoul Korea, Republic of (South)

## Abstract

**Background:**

Women’s elevated risk of Alzheimer’s disease (AD) compared to men remains unclear, with gonadal hormones proposed as potential contributors. This study aimed to explore the association between follicle‐stimulating hormone (FSH), estradiol (E2), neuropsychological AD stages, and cerebral Aβ deposition.

**Methods:**

A total of 679 subjects were included in the study (N = 198 for cognitively normal (CN), N = 373 for mild cognitive impairment (MCI), and N = 108 for AD dementia groups). The inclusion criteria for all subjects were: (1) female sex, (2) age ≥ 55 years, (3) menopause defined as a minimum of 12 months passed since the last menstruation. Amyloid‐PET was conducted using ^18^F‐flutemetamol (^18^F‐FMM).

**Result:**

Results revealed significant differences in serum FSH levels among CN, MCI, and AD dementia groups (P < 0.001 for ANOVA), persisting after adjusting for age (P < 0.01 for ANCOVA). Post‐hoc analysis indicated higher FSH levels in the AD dementia group compared to MCI and CN groups, with no significant FSH differences between CN and MCI (Figure 1). Conversely, serum E2 levels showed no group differences (P = 0.076 for ANOVA), remaining statistically insignificant after age adjustment (P > 0.1 for ANCOVA). Correlation analysis demonstrated a negative association between FSH levels and CERAD‐K total score, including all sub‐measures except for constructional praxis. No significant associations were found between E2 levels and neuropsychological profiles. In the subset of 311 participants undergoing ^18^F‐FMM PET analysis, the A‐PET+ group exhibited higher FSH levels than A‐PET‐ group, while E2 levels did not differ (Figure 2). Positive correlations were observed between FSH levels and global and regional SUVR in the PCC/PC, frontal lobe, parietal lobe, and lateral temporal lobe (Figure 4). However, E2 levels showed no significant association with cerebral Aβ deposition.

**Conclusion:**

This study provides insights into the link between FSH, neuropsychological AD stages, and cerebral Aβ deposition in post‐menopausal women, highlighting FSH’s potential role as a biomarker. In contrast, such associations were not observed for E2. Further research is needed to unravel the underlying mechanisms and explore therapeutic avenues targeting hormonal pathways in AD.